# The efficacy of risk scores for predicting abdominal wound dehiscence: a case-controlled validation study

**DOI:** 10.1186/1471-2482-14-65

**Published:** 2014-09-02

**Authors:** Jakub Kenig, Piotr Richter, Anna Lasek, Katarzyna Zbierska, Sabina Zurawska

**Affiliations:** 1 3rd Department of General Surgery, Jagiellonian University Medical College, Krakow, Poland

**Keywords:** Abdominal wound dehiscence, Risk score, Abdominal complications

## Abstract

**Background:**

The medical literature includes two risk scores predicting the occurrence of abdominal wound dehiscence. These risk indices were validated by the authors on the populations studied. However, whether these scoring systems can accurately predict, abdominal wound dehiscence in other populations remains unclear.

**Methods:**

A retrospective analysis was performed using the medical records of patients treated at a tertiary-care teaching hospital between 2008 and 2011. Patients that underwent laparotomy procedures complicated by the development of postoperative abdominal wound dehiscence were included into the study. For each of the cases, three controls were selected.

**Results:**

Among the 1,879 patients undergoing intra-abdominal, 56 patients developed wound dehiscence and 168 patients included in the control group. Calculation of risk scores for all patients, revealed significantly higher scores in the abdominal wound dehiscence group (p < 0.001). The median score was 24 (range: 3–46) and 4.95 (range: 2.2-7.8) vs.10 (range:-3-45) and 3.1 (range:0.4-6.9), for the Veterans Affairs Medical Center (VAMC) and Rotterdam abdominal wound dehiscence risk score in the dehiscence and control groups, respectively. The area under the curve, on the ROC plot, was 0.84 and 0.76; this confirmed a good and moderate predictive value for the risk scores. The fit of the model was good in both cases, as shown by the Hosmer and Lemeshow test.

**Conclusions:**

Both the VAMC and Rotterdam scores can be used for the prediction of abdominal wound dehiscence. However, the VAMC prognostic score had better calibration and discriminative power when applied to the population in this study and taking into consideration our method of control selection.

## Background

Abdominal wound dehiscence is one of the most serious postoperative complications; the incidence in the adult population is reported as 0.3-3.5%, and among the elderly it is as high as 10%. In about 20-45% of cases, evisceration becomes a significant risk factor, which is associated with death during the perioperative period [1,2]. Several publications have indentified risk factors associated with this complication; however, many of the reports have conflicting results. Two papers, by van Ramshorst GH et al. in 2010 and Webster C et al. in 2003, reported on a scoring system that was developed based on multivariable stepwise logistic regression models of preoperative, intraoperative and postoperative variables that were entered sequentially as independent predictors of wound dehiscence. Both risk indices were validated by the authors of these studies based on the populations studied; they aided clinical management [3,4]. However, whether these scoring systems can accurately predict abdominal wound dehiscence in other populations remains unclear.

## Methods

### Selection of patients

A retrospective analysis was performed using the medical records of patients treated at a tertiary-care teaching hospital from January 2008 to December 2011. The study included a group of patients that underwent laparotomy procedures complicated by postoperative abdominal wound dehiscence, defined as "separation of the layers of the surgical wound, partial or complete, with disruption of the fascia". The study used case–control methodology with reversed-flow design. For each case, three controls were selected that had laparotomy procedures, were matched for a similar period of time (time interval from 1 day to 1 month), age (±2-3 years), gender, diagnosis of underlying disease, and type of surgery performed. Patients that had open abdomen procedures were excluded.

### Data collection

Preoperative patient characteristics including risk factors, intra- and peri- operative processes of care, and postoperative adverse occurrences were recorded based on both electronic and paper medical records. Other data such as laboratory values and culture results were pulled into the dataset from other computerized sources. Particular emphasis was placed on the analysis of the factors described in both publications mentioned above.

### Definition of factors used in this study

1. Wound infection occurrence was recorded in the database when at least one of the following was observed within 30 days after the operation: purulent drainage (culture documentation not required), organisms are isolated from fluid/tissue, at least one sign of inflammation (pain or tenderness, induration, erythema, local warmth of the wound), the wound was deliberately opened by the surgeon or the surgeon declared the wound infected.

2. Anemia: a blood hemoglobin level less than 12 g/dl

3. Jaundice: the total level of bilirubin in blood serum 3 mg/dl or higher.

4. Postoperative coughing was defined as coughing documented by nurses in the patient charts before the diagnosis of abdominal wound dehiscence, or before discharge in patients without abdominal wound dehiscence.

5. Ascites - accumulation of fluid in the peritoneal cavity on clinical examination and/or visible on ultrasound.

### Prognostic scores

The two published abdominal wound dehiscence risk indices were used in this study. The first one was based on data from the Veterans Affairs National Surgical Quality Improvement Program (NSQIP) used at 132 Veterans Affairs Medical Centers between October 1996 and September 2000, hereinafter referred to as the VAMC risk score. The second index was based on medical registers developed from January 1985 to December 2005 at an academic teaching hospital from the Netherlands, hereinafter referred to as the Rotterdam risk score. Detailed information on these indices can be found in the relevant publications [3,4]. For both indices a higher value predicts a higher risk.

### Missing data

Patients with missing data associated with risk factors of interest were excluded from the study. For the rest of the patients included in the final analysis, data were missing for 3% of the patients in the dehiscence group and 2% in the control group.

### Statistical analysis

Qualitative and quantitative data were used to describe the study results. Quantitative parameters are expressed as the mean value ± standard deviation or median (range) as appropriate. The remaining cases were summarized as counts and percentages. The data were analyzed using the Statistica 10.0 software suite (StatSoft). The Shapiro-Wilk W and the Kolmogorov-Smirnov tests, with the Killiefors correction, were used to verify the normality of the distribution of the results. Based on these analyses, the data were analyzed using parametric or non-parametric tests.

Validation of the scores was performed using standard tests to measure calibration and discrimination. The discriminatory ability of the scores in predicting abdominal wound dehiscence was measured using the area under the receiver operating characteristic curve (AUC). Sensitivity, specificity, positive predictive value (PPV), and negative predictive value (NPV) were calculated using standard formulas for each score. A comparison between the AUCs was performed using the *χ*^2^ test. The calibration of the predicted to the observed development of abdominal wound dehiscence was measured using the Hosmer-Lemeshow goodness-of-fit statistic for 10 covariate groups, whenever possible. Statistical significance was defined as a two-sided p ≤ 0.05.

The retrospective access to the database has been approved by the review board of the 3^rd^ Department of General Surgery Jagiellonian University Medical College.

## Results

### Patient characteristics

Among the 1,879 patients undergoing intra-abdominal procedures during the study period 56 patients were included in the validation group; the patients developed wound dehiscence during the postoperative period and represented 2.9% of all operations performed. The group consisted of 37 men and 19 women; there was a statistically significant difference with regard to gender, men accounted for more of the cases (p = 0.034). The mean age was 66.8 ± 12.6 years. Abdominal wound dehiscence occurred on average at the 9.8 ± 6.5 postoperative day (median: 8 days). The mortality of patients in this group was 25%. In addition, more patients were operated on as a emergency procedures 45 (80.4%) vs. 11 patients in the elective group, this difference was statistically significant (p <0.001). There were 168 patients in the control group based on the above mentioned criteria. The baseline characteristics of the patients are reported in Table 1.

**Table 1:** Baseline characteristics of patient from the dehiscence and control group

**Factor**	**Study group**	**Control group**	**P value**
Number (male/female):	56 (37/19)	168 (95/73)	0.210
Age [years]	66.6 ± 13	66.8 ± 12.8	0.872
Type of the procedure [n]:			0.276
- Elective	11 (20%)	44 (26%)	
- Emergency	45 (80%)	124 (74%)	
Type of operation [n]:			
- Stomach/duodenum	5 (9%)	14 (8%)	0.898
- Gall bladder	5 (9%)	13 (8%)	0.794
- Small intestine	9 (16%)	36 (21%)	0.475
- Large intestine	27 (49%)	83 (49%)	0.928
- Others	10 (16%)	22 (13%)	0.450
Opening of the bowel [n]	42 (76%)	122 (73%)	0.163
Malignancy [n]	24 (44%)	71 (42%)	0.858
Co-morbidities [n]:			
- Hypertension	28 (51%)	92 (55%)	0.619
- Heart disease	23 (42%)	61 (36%)	0.464
- Diabetes	8 (14%)	28 (17%)	0.711
- COPD	9 (16%)	16 (9%)	0.163
- Other	42 (76%)	116 (69%)	0.326
BMI:<20.5/20.5-30/>30 kg/m^2^ [n]	12/31/13	24/119/24	0.123
Past operations [n]	25 (45.5%)	91 (54%)	0.261
Smoking [n]	15 (27.3%)	41 (24%)	0.670
**Chronic steroids use [n]**	**7 (12.7%)**	**6 (4%)**	**0.011**
**Wound infection**	**34 (61%)**	**23 (14%)**	**<0.001**
Anastomotic insufficiency	5 (9%)	5 (3%)	0.768
**Circulatory insufficiency [n]**	**21 (37%)**	**43 (26%)**	**0.042**
Median laparotomy [n]	47 (84%)	143 (85%)	0.952
Retention sutures [n]	13 (23%)	27 (16%)	0.173
Time of op. [n]:			
7–15.00	24 (43%)	81 (48%)	0.681
15.01-23.59	26 (46%)	73 (43%)	0.729
00.00-6.59	6 (11%)	14 (8.3%)	0.890
**ICU admission [n]**	**25 (45%)**	**39 (23%)**	**0.001**
**Length of hospital. [days]**	**38.3 ± 27.1**	**15.8 ± 12.9**	**<0.001**
Biochemical factors:			
- WBC [10^3^/μl]	12.5 ± 6.0	11.6 ± 5.9	0.260
- HCT [%]	35.9 ± 7.2	37.7 ± 6.4	0.096
- HGB [g/dl]	12.2 ± 4.6	12.4 ± 2.2	0.669
- CRP [mg/l]	127.13 ± 108.2	111.49 ± 100.27	0.476
- Albumin [g/l]	30.2 ± 12.9	32.69 ± 9.6	0.260
- Protein [g/l]	52.44 ± 13.9	56.85 ± 13.8	0.167
- Creatinine [μmol/l]	104.4 ± 62.5	91.98 ± 63.7	0.308
Time to dehiscence [days]	9.8 ± 6.5	-	

The patients that developed abdominal wound dehiscence had a higher rate of wound infection, circulatory insufficiency, increased length of hospitalization and were more likely admitted to the ICU; these differences were statistically significant. The other factors studied did not show statistically significant differences.

### Comparison of predicted dehiscence risk

Calculation of risk scores for all patients revealed significantly higher scores in both abdominal wound dehiscence groups (p < 0.001). The median scores were 24 (range: 3–46) and 4.95 (range: 2.2-7.8) vs. 10 (range: -3-45) and 3.3 (range: 0.4-6.9), for the VAMC and Rotterdam abdominal wound dehiscence risk score in the dehiscence and control groups, respectively (p < 0.001). Tables 2 and 3 are showing the VAMC and Rotterdam scores variables and characteristics of the validation population.

**Table 2:** VAMC score variables and characteristics of the validation population

**Variables of the VAMC score**	**Abdominal wound dehiscence [n = 56]**	**No abdominal wound dehiscence [n = 168]**	**P value**
**CVA/stroke no deficit**	9%	5%	0.280
**History COPD**	16%	9%	0.163
**Current pneumonia**	12%	4%	0.081
**Emergency procedure**	80%	74%	0.715
**Operative time >2.5 h**	37%	40%	0.834
**PGY4**	23%	25%	0.833
**Clean wound classification**	23%	27%	0.683
**Superficial wound infection**	21%	10%	0.053
**Deep wound infection**	39%	4%	<0.001
**Failure to wean**	21%	16%	0.448
**One or more complications**	70%	42%	0.032
**Return to OR**	0%	0%	1.000

**Table 3:** Rotterdam score variables and characteristics of the validation population

**Variables of the Rotterdam score**	**Abdominal wound dehiscence [n = 56]**	**No abdominal wound dehiscence [n = 168]**	**P value**
**Age category**			
**40**–**49**	12%	11%	0.744
**50**–**69**	48%	45%	0.815
**>70**	39%	44%	0.691
**Male gender**	66%	56%	0.502
**Chronic pulmonary disease**	16%	9%	0.163
**Ascites**	4%	0%	0.110
**Jaundice**	7%	4%	0.399
**Anemia**	61%	16%	<0.001
**Emergency surgery**	80%	74%	0.715
**Type of surgery**			
**Gallbladder/bile duct**	9%	8%	0.794
**Esophagus**	0%	0%	1.000
**Stomach and duodenum**	9%	8%	0.794
**Small bowel**	16%	21%	0.520
**Large bowel**	49%	49%	0.964
**Vascular**	0%	0%	1.000
**Coughing**	21%	3%	<0.001
**Wound infection**	61%	14%	<0.001

The relationship between all scores was statistically significant. The area under the curve, in the ROC plot, was 0.84 and 0.76 respectively, showing a good and moderate predictive value of the risk scores (Table 4). However, the VAMC score more successfully predicted patients that would develop dehiscence.

**Table 4:** Area under ROC curve for each score (area+/-SE) and the odds ratio (OR) as a risk coefficient for the abdominal wound dehiscence score and dehiscence

	**AUC**	**SE**	**P value**	**OR**	**95% CI for OR**
**VAMC score**	0.84	0.03	<0.001	1.1	1.1-1.2
**Rotterdam score**	0.76	0.04	<0.001	2.2	1.7-2.9

AUC – area under the curve (ROC), OR – odds radio, SE – standard error of area, type I error probability of area.

The odds ratio as risk coefficient was examined using a binary logistic regression model. When the VAMC and Rotterdam scores increased by one unit the predicted odds changed by a multiplicative factor of 1.1 and 2.2, respectively. This indicates that for an increase of 1 point, on both risk scores, the risk of abdominal wound dehiscence increases 1.1 and 2.2 times (Table 4).

The efficacy of both scores for predicting abdominal wound dehiscence during the postoperative period can be defined as sensitivity and its efficacy in predicting a complication free course (in terms of dehiscence) defined as specificity. The values of the VAMC and Rotterdam scores are shown in Table 5. These results are presented at optimal cut off values and at the values used in primary publications. In the calculations, two types of errors of abdominal wound dehiscence scores were investigated. These can be referred to as the false positive and negative results (Table 5).

**Table 5:** The correct and misclassification error probabilities (%) of both abdominal wound dehiscence risk score at optimal cut off values and at values used in primary publications

	**Specificity (%)**	**Sensitivity (%)**	**False + (%)**	**False – (%)**	**PPV (%)**	**NPV (%)**	**Accuracy (%)**	**Cut off value**
**VAMC score**	94%	48%	5%	50%	75%	85%	83%	25 points
	70%	82%	29%	18%	48%	92%	73%	14 points*
**Rotterdam score**	98%	20%	2%	80%	73%	78%	78%	5.8 points
	71%	73%	29%	27%	45%	89%	71%	3.8 points*

*Cut off values used in primary publications.

### Comparison of discrimination

Table 6 shows that the overall area under the receiver operating characteristic curve for wound dehiscence was 0.84 and 0.76, for VAMC and Rotterdam scores, respectively. The AUC under the VAMC curve was significantly higher than the Rotterdam curve (p < 0.001), indicating a better discriminatory ability.

**Table 6:** Comparing discriminatory ability and calibration among various scores for the wound dehiscence

**Risk score**	**Are under ROC curve**	**Hosmer-Lemeshow (9 groups)**	**P value**
**VAMC score**	0.84	6.75	0.461
**Rotterdam score**	0.76	12.4	0.083

### Calibration of prediction scores

The fit of the model was good in both cases, as shown by the Hosmer and Lemeshow test (p = 0.461 and p = 0.083, respectively, as it is shown in Table 6). However, in the case of the VAMC score the calibration was significantly better.

## Discussion

Prior studies have identified several risk factors associated with the development of abdominal wound dehiscence, such as: age (>65 years old), gender (male), smoking, obesity, chronic steroid therapy, anemia, jaundice, uremia, diabetes, low albumin level, chronic obstructive pulmonary disease (COPD), cancer, wound infection, and emergency surgery [5-11]. The results of this study indicate that wound dehiscence is a complex process that is influenced by factors both of a general and local nature, as well as pre-, intra-and postoperative timing. Only the common occurrence of a number of factors lead to the development of this complication. Most of the risk factors do not depend directly on the surgeon, but rather on patient factors such as: gender, age, type of disease to be treated, mode of surgery, and chronic steroid use. No significant differences were observed between the study and control groups with regard to diabetes, COPD, anemia, uremia, jaundice, and the albumin levels. However, consistent with the findings of other publications, the most important risk factor for the development of abdominal wound dehiscence was a surgical site infection.

Scoring systems are designed to estimate the probability of occurrence of an undesired event. Such systems can be used to aid clinical management, resource allocation and quality assessment. There are only two scoring systems in the medical literature that are used for determining the risk for developing an abdominal wound dehiscence; the populations studied validated both. However, external validation is essential before a scoring system is applied to a group of patients different from the one originally used for model development.

This is the first study to compare the validity of the reported indices in our population. Both the VAMC and Rotterdam scores can be used to predict abdominal wound dehiscence. The relationships between all scores were statistically significant and the area under the curve of the ROC plot showed a good (0.84) and moderate (0.76) predictive value. However, in this study the VAMC score showed a significantly better discriminatory ability. Moreover, the VAMC score had better calibration compared to the Rotterdam score. This is due the fact that the Rotterdam score consists of many variables that our control group was matched for (age, gender, emergency surgery, type of surgery). Among the variables, only the VAMC score included the risk factor of emergency procedures. In addition, the population studied here may be more similar to the population in the VAMC study with more co-morbidities than the general population. Furthermore, the Rotterdam score was designed to avoid excessive inclusion of emergency operations in the control group. The population assessed in this study, was more like the VAMC population with regard to the number of emergency operations; which were significantly higher compared to elective procedures. In the study by Gomez Diaz et al., authors also concluded that the Rotterdam score has same limitations in the preoperative assessment and additional refinements are needed to improve accuracy. This is mainly due to the fact that its comprises a list of postoperative factors, including, the key factor in the assessment, surgical wound infection [12].

The limitations of this study include the following. The design was a retrospective analysis. However, the data were validated as thoroughly as possible. In addition, the data is from a single centre, which limits the generalization of the findings. The use of matching cases and controls could have affected the sensitivity and specificity of tests. However, the study was designed to reduce the number of confounding variables. Both scores could be used to distinguish patients with a high risk for abdominal wound dehiscence that had similar disease and type of surgery.

## Conclusion

In conclusion, both the VAMC and Rotterdam score indices can be used to predict the development of abdominal wound dehiscence. The VAMC prognostic score had better calibration and discriminative power when applied to the population evaluated in this study and taking into consideration our method of control selection. Moreover, these scores can be used to distinguish patients at high risk for developing abdominal wound dehiscence that have similar diseases and undergo similar surgical procedures.

## Competing interests

The authors declare that they have no competing interests.

## Authors’ contributions

JK, Study conception and design, Analysis and interpretation of data, Acquisition of data Drafting of manuscript. PR: Study conception and design, Drafting of manuscript, Critical revision of manuscript. AL: Drafting of manuscript, Acquisition of data. KZ: Acquisition of data. SZ: Acquisition of data. All authors read and approved the final manuscript.

## Pre-publication history

The pre-publication history for this paper can be accessed here:

http://www.biomedcentral.com/1471-2482/14/65/prepub
